# Optimization of Cultural Conditions for Protease Production by a Fungal Species

**DOI:** 10.4103/0250-474X.65017

**Published:** 2010

**Authors:** P. Kamath, V. M. Subrahmanyam, J. Venkata Rao, P. Vasantha Raj

**Affiliations:** Department of Pharmaceutical Biotechnology, Manipal College of Pharmaceutical Sciences, Manipal University, Manipal-576 104, India

**Keywords:** Enzyme production and optimization, fungal protease, tyrosine production

## Abstract

Studies were carried out on a paddy soil fungal isolate identified to be a strain of *Aspergillus niger* from Manipal. The parameters that largely impact enzyme production viz., fermentation time, impeller speed, pH, temperature and nutrient supplements were studied. Optimization of production parameters for production of protease was done by the single-parameter mode. Casein served as substrate and proteolytic activity was estimated using Folin-Ciocalteau method at 660 nm. A maximum yield of 71.3 mg tyrosine/g casein substrate was produced in 96 h on a soluble starch medium at pH 4 in shake flask experiments. Production was carried out on a 3-liter fermenter and 40.7 mg of tyrosine was liberated/g of substrate. The enzyme was extracted with 50% ammonium sulfate and sodium dodecyl sulfate-Polyacrylamide gel electrophoresis showed two bands having mw 45.7 kDa and 38.5 kDa, respectively. The enzyme activity was found to be 147.84 U/ml.

Proteases account for nearly 60% of the industrial enzyme market and have wide applications in many industries *viz*., textiles, detergents, food processing especially for cheese ripening, meat tenderizing, animal nutrition, pharmaceuticals, paper industry and food industry[1]. These enzymes are also reported to have a significant role in development and manifestation of dreadful diseases such as AIDS and cancer[2]. Although extensive work has been reported using fungal strains, still work on isolation and characterization is being carried out to isolate microorganisms having better activity and clinical importance[3]. The present study was carried out on a promising protease producing fungal isolate from paddy field soil of Manipal.

## MATERIALS AND METHODS

The parameters that largely impact enzyme production are fermentation time, impeller speed, pH and fermentation temperature and nutrient supplements. In the present study, an attempt was made to optimize the production parameters for a fungal isolate (PF_1_) isolated from paddy soil, extract the enzyme and to determine its molecular size using sodium dodecyl sulfate polyacrylamide gel electrophoresis (SDS-PAGE). This isolate was grown and maintained on potato dextrose agar (PDA) slants at 28° for 10 days and the slants were stored at 4°.

### Identification of the culture:

The fungal isolate was subjected to some gross morphological and biochemical studies *viz*., gelatin liquefaction, casein hydrolysis, tyrosine utilization and carbohydrate utilization[4]. Gelatin was added at 15% level to sterile nutrient broth tubes, inoculated with the fungal isolate and incubated at 28° for 48 h. After incubation, the tubes were kept at a temperature of 2-3° for 1h and tested for liquefaction if any. PDA, supplemented with 20% skimmed milk, was used for casein hydrolysis and incubated at 25° for 96 h for the organism. ISP medium no. 7 was used for tyrosine utilization test. Inoculated slants were kept for incubation for 10 to 14 days at 28° and observed for brown black or greenish brown diffusible pigment. Each of the eight sugars *viz*., glucose, arabinose, xylose, cellulose, fructose, sucrose, raffinose and maltose was employed for testing the carbohydrate utilizing ability of PF_1_. Sugars (at 1% level) were added to the ISP medium no. 9, inoculated and incubated at 28° for 2 weeks.

### Batch process:

Spore suspension from a 10-day old slant (10^5^-10^6^ colony forming units/ml) was added to an inoculation medium containing glucose (2.0%), yeast extract (1.0%), K_2_HPO_4_ (0.1%), KH_2_PO_4_ (0.1%), MgSO_4_ (0.02%) and distilled water q.s. with pH initially adjusted to 7.0 and incubated at 150 rpm at 28°. Production medium consisted of glucose (2.0%), yeast extract (1.0%), K_2_HPO_4_ (0.1%), KH_2_PO_4_ (0.1%), casein (1.5%) at pH 7.0 was inoculated at 10% level and maintained at 150 rpm on an orbital shaking incubator (Remi) for 7 days at 28°. The amount of tyrosine produced was estimated from 24 h samples by Folin and Ciocalteau method at 660 nm[5].

### Optimization of production parameters:

The protocol adopted for optimization of process parameters aimed to evaluate the effect of an individual parameter at a time and to incorporate it at the standard level before optimizing the next parameter. Various process parameters influencing protease production *viz*., casein concentration (0.5-2.5%), fermentation time (studied up to 7 days with 24 h sampling), fermentation temperature (20-40°), initial pH (3-8) and agitation speed (50-250 rpm) were studied. The effect of medium supplements based on carbon (dextrose, sucrose, fructose, starch and lactose), organic nitrogen (malt extract, peptone, tryptone, and yeast extract) and inorganic nitrogen (ammonium sulphate, ammonium chloride, potassium nitrate and ammonium citrate) sources and minor elements magnesium sulphate and calcium carbonate on enzyme production was studied by incorporating the constituent/parameter individually in the production medium.

### Fermentation under optimized conditions:

Fermentation was carried out using a 3 l fermenter (Scigenics, India) set under optimized conditions of substrate concentration, time, temperature, pH, rpm and nutrient supplements with an aeration rate of 1.0 vvm.

### Enzyme extraction:

After complete fermentation, the broth was centrifuged at 10000 rpm for 10 min at 4° to remove mycelium. The supernatant was added with ammonium sulphate to obtain 50% saturation for the precipitation of the enzyme. The precipitated proteins were pelletized by centrifugation at 8000 rpm at 4° for 20 min. The protein pellet was then suspended in 5 ml 0.1 M Tris- HCl buffer pH 7.8 and stored at —20°.

### Determination of molecular weight:

The molecular weight of the crude enzyme of isolate PF_1_ was determined by SDS-PAGE using 10% acrylamide. The gel was stained with Coomassie brilliant blue solution and destained by washing gel with acetic acid/ methanol solution. After destaining, the destained gels were viewed with the help of Alphaimager (JH BIO) software for determination of molecular weight by comparing with a standard protein marker.

### Determination of enzyme activity:

Protease activity was determined using 0.6% casein substrate at pH 4.0 and 37°. After incubation for 10 min, the reaction was stopped using trichloroacetic acid (TCA) and the amount of liberated amino acid was measured at 660 nm using a tyrosine standard. One unit (U) is defined as the amount of enzyme which yields 1 µg of tyrosine per min at 37° and pH 4.0.

## RESULTS AND DISCUSSION

**Fig. 1:**
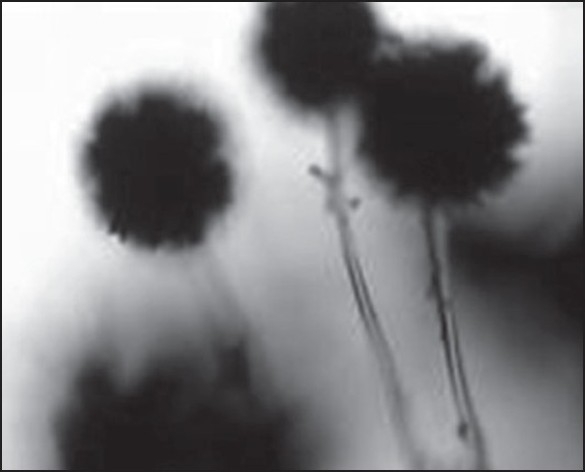
Conidiophore of the fungal isolate. Conidiophore originated from the basal foot cell located on supporting hyphae and terminated in a vesicle at the apex

The isolated organism was initially white on potato dextrose agar and on sporulation turned black. The reverse color was pale yellow and the colonies showed a compact yellowish white basal felt covered by a dense layer of brownish black conidial heads. The conidiophores originated from the basal foot cell located on supporting hyphae and terminated in a vesicle at the apex (fig. 1). The organism was found to liquefy gelatin, exhibited caseinolytic activity and did not show any tyrosinase activity (as no brown, black or greenish brown pigment was observed). The organism utilized (Table 1) glucose, fructose, sucrose and raffinose well, while maltose and xylose were poorly utilized and could not utilize arabinose and cellulose. The fungus formed black spores from biseriate phialides which is the major distinction currently separating *A. niger* from other species of *Aspergillus*. Colony color of various *Aspergillus* species[6] is given in Table 2. The isolate was identified by Agharkar Research Institute, Pune. The isolate belonged to *A. niger* group of microorganism. Since the organism appeared promising, experiments were devised to optimize the cultural conditions.

**TABLE 1:** CARBOHYDRATE UTILIZATION

Glucose	Raffi nose	Arabinose	Xylose	Cellulose	Fructose	Maltose	Sucrose
+ + (Extensive growth)	+ + (Extensive growth)	‐ (No growth)	+ (Moderate growth)	‐ (No growth)	+ + (Extensive growth)	+ (Moderate growth)	+ + (Extensive growth)

Each of the eight sugars viz., glucose, arabinose, xylose, cellulose, fructose, sucrose, raffi nose and maltose was employed for testing the carbohydrate utilizing ability of PF1. Sugars (at 1% level) were added to the ISP medium no. 9, inoculated and incubated at 28° for two weeks.

**TABLE 2:** MORPHOLOGICAL STUDIES

Species	Surface	Reverse
A. *clavatus*	Blue-green	White, brownish with age
A. *flavus*	Yellow-green	Goldish to red brown
A. *fumigatus*	Blue-green to grey	White to tan
A. *glaucus*	Green with yellow areas	Yellowish to brown
A. *nidulans*	Green, buff to yellow	Purplish red to olive
A. *niger*	Black	White to yellow
A. *terreus*	Cinnamon to brown	White to brown
A. *versicolor*	White at the beginning, turns to yellow, tan, pale green or pink	White to yellow or purplish red
PF1 isolate	Black	Pale yellow

The isolate was grown on Potato dextrose agar at 28° for 10 days, and observed after sporulation.

Initially the production was studied using casein as substrate. Highest proteolytic activity was seen after 120 h at 1.5% casein concentration (Table 3) and the amount of liberated tyrosine reached 40.3 mg/g substrate. It was reported that casein induced protease production at low concentrations and served as nitrogen metabolite repressor at high concentration in many types of yeast[7].

**TABLE 3:** PRODUCTION PARAMETERS- SUBSTRATE, TEMP, pH AND RPM

Parameters	Tyrosine (mg)/ gram of substrate Time (h)							
	24	36	48	72	96	120	144	168
Substrate concentration %								
0.5	4.6	14.4	16.4	17.6	18.8	26.8	20.0	18.6
1.0	5.7	7.6	7.8	24.6	26.3	36.6	34.0	30.3
1.5	5.4	11.8	20.8	27.4	31.6	40.3	39.5	36.6
2.0	4.8	9.6	13.5	28.6	31.1	38.1	37.6	35.2
2.5	4.9	10.1	12.6	20.9	23.6	33.1	30.6	29.0
Fermentation temperature °								
20			4.5	6.4	12.9	22.8	20.1	
25			22.8	32.8	44.2	55.1	49.3	
28			24.5	36.2	51.2	62.5	59.3	
35			20.6	28.9	42.4	55.6	50.9	
40			10.7	19.5	32.7	36.8	33.8	
MEDIUM pH								
3	5.5		24.5	35.2	45.3	59.2	56.3	55.1
4	6.2		25.9	37.7	48.2	61.4	58.0	54.3
5	5.1		22.7	36.1	44.4	56.2	54.1	52.3
6	5.4		23.1	29.4	38.5	44.2	34.5	32.1
7	4.1		19.4	25.5	32.9	42.3	40.1	38.6
8	2.5		15.3	20.1	26.5	22.7	24.5	22.1
RPM								
50			5.9	12.6	15.4	18.9	17.8	
100			16.5	24.9	35.5	40.1	38.4	
150			24.0	35.5	51.2	61.4	57.5	
200			22.8	33.3	52.4	53.6	51.2	
250			22.4	37.6	49.6	45.8	44.1	

Various process parameters infl uencing protease production viz., casein concentration (0.5-2.5%), fermentation time (studied up to 7 days with 24 h sampling), fermentation temperature (20-40°), initial pH (3-8) and agitation speed (50-250 rpm) were studied.

Temperature plays an important role in the synthesis of enzymes. Maximum proteolytic activity was found at temperature of 28° at 120 h. The isolate was mesophilic in nature. The organism produced maximum protease at pH 4. pH had a major effect on the morphology of the organism. At alkaline pH, the organism formed smaller pellets which were rigid, whereas in the case of acidic pH the pellets were bigger in size and fluffy. Donnell *et al*., have reported that *Aspergillus niger* acidified the medium and the pH came down to 3 after two days of fermentation[8]. In the present study it was observed that there was a fall in the pH after two days of fermentation, with a large decline in activity at pH above neutral. From the pH profile it was found that the fungal organism produced acid protease. Agitation helps in the proper mixing of the nutrients and plays a major role in growth of microorganism. A speed of 150 rpm was found to be optimum. Almost all the carbon sources (Table 4) enhanced protease production and maximum proteolytic activity was observed with 1% soluble starch. This meant that the organism could secrete extracellular amylase to hydrolyse the starch in the medium. The results revealed that soluble starch 1% was the best carbon source when compared to dextrose 2% for protease production. A maximum of 62.3 mg tyrosine liberated/g of substrate was produced in 96 h. So there was a reduction in the fermentation time for maximum protease production. Negi and Banerjee found that 1% starch is a good inducer for the production of protease[1]. The present isolate PF_1_ was isolated from paddy field soil which has starch residues. Hence, addition of starch can simulate the natural conditions for the isolate. When compared with other nitrogen supplements, peptone was the best organic nitrogen source. Rahman reported that peptone and casein were good inducers of proteases and peptone at 1% level gave best yield[9].

**TABLE 4:** PRODUCTION PARAMETERS- CARBON, NITROGEN AND METAL IONS

Parameters	Tyrosine (mg)/ gram of substrate Time (h)				
	48	72	96	120	144
Carbon supplements 1%					
Lactose	21.2	31.7	45.4	48.7	46.2
Dextrose	16.6	27.5	39.4	44.5	41.3
Fructose	23.7	39.5	57.3	54.1	50.3
Sucrose	20.2	32.7	43.7	52.3	48.3
Soluble Starch	25.4	42.7	62.3	61.1	59.5
Organic nitrogen supplements 1%					
Malt extract	12.4	22.9	34.5	35.4	32.1
Peptone	24.3	43.2	63.8	61.9	56.7
Tryptone	23.2	40.2	52.3	52.6	49.2
Yeast extract	21.2	32.4	45.1	47.9	44.7
Inorganic nitrogen supplements 1%					
Ammonium sulphate	24.1	44.5	65.2	65.7	62.3
Ammonium chloride	25.4	44.1	64.4	63.5	62.7
Potassium nitrate	26.7	45.9	67.9	64.7	64.2
Ammonium citrate	23.4	43.8	64.3	64.0	62.2
Effect of metal ions					
Flask 1 (0.01%MgSO_4_ and 0.1%CaCO_3_)	26.4	46.9	68.8	68.1	67.3
Flask 2 (0.02%MgSO_4_ and 0.02%CaCO_3_)	25.3	42.5	66.7	65.5	64.3
Flask 3 (0.1%MgSO_4_ and 0.01%CaCO_3_)	26.1	45.5	67.4	66.6	66.2
Flask 4 (0.05%MgSO_4_ and 0.05%CaCO_3_)	24.4	44.7	67.0	67.4	65.9
Flask 5 (0.1%MgSO_4_ and 0.1%CaCO_3_)	25.7	47.3	71.3	68.8	66.4

The effect of medium supplements based on carbon (dextrose, sucrose, fructose, starch and lactose), organic nitrogen (malt extract, peptone, tryptone, and yeast extract) and inorganic nitrogen (ammonium sulphate, ammonium chloride, potassium nitrate and ammonium citrate) sources and minor elements magnesium sulphate and calcium carbonate on enzyme production was studied by incorporating the constituent/parameter individually in the production medium

All the inorganic nitrogen sources showed enhancement in the protease production. Potassium nitrate showed the maximum effect. Shumi *et al*. have reported that *Aspergillus funiculosus* produced maximum protease when supplemented with potassium nitrate[10]. There was increase in tyrosine levels with calcium carbonate and magnesium sulphate. Maximum amount of 71.3 mg/g substrate was liberated in 96 h on a medium, consisting starch (1%), casein (1.5%), KNO_3_ (1%), MgSO_4_ (0.1%) and CaCO_3_ (0.1%) with pH 4 at, 28°.

**Fig. 2:**
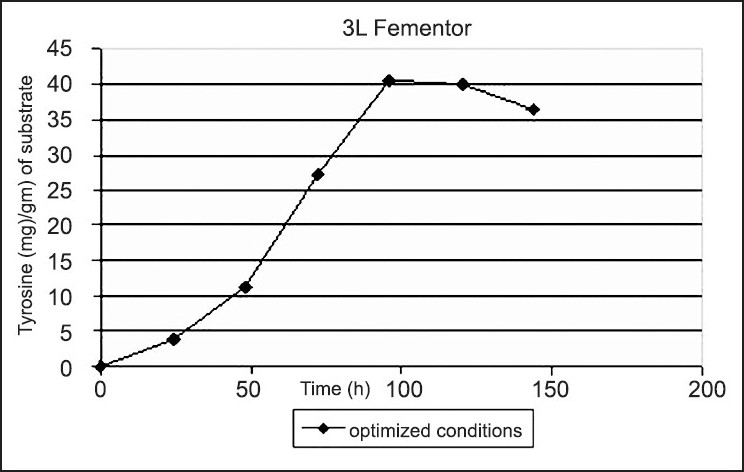
Fermentation under optimized conditions. Fermentation was carried out using a 3 l bioreactor and the amount of tyrosine liberated was estimated every 24 h

After optimizing the production parameters, fermentation was carried out under optimized conditions on a 3 l Bioreactor. The amount of tyrosine liberated/g of substrate was found to be 40.7 mg (Table 5 and fig. 2). Although the amount of tyrosine produced is lower than that produced in the shake flask experiments, the time remained same i.e.; at 96 h for maximum production, with other operating parameters remaining the same. The lower production could be due to increase in volume of the medium and lower aeration level. Olajuyigbe *et al*., reported acid protease synthesis by *Aspergillus niger* (NRRL 1785). Maximum enzyme synthesis is obtained at 96 h of fermentation[6].

**TABLE 5:** FERMENTATION UNDER OPTIMIZED CONDITIONS

Production of tyrosine (mg)/g of substrate in h					
24	48	72	96	120	144
3.8	11.3	27.1	40.7	39.9	36.3

Fermentation was carried out using a 3 l bioreactor and the amount of tyrosine liberated was estimated every 24 h

**Fig. 3:**
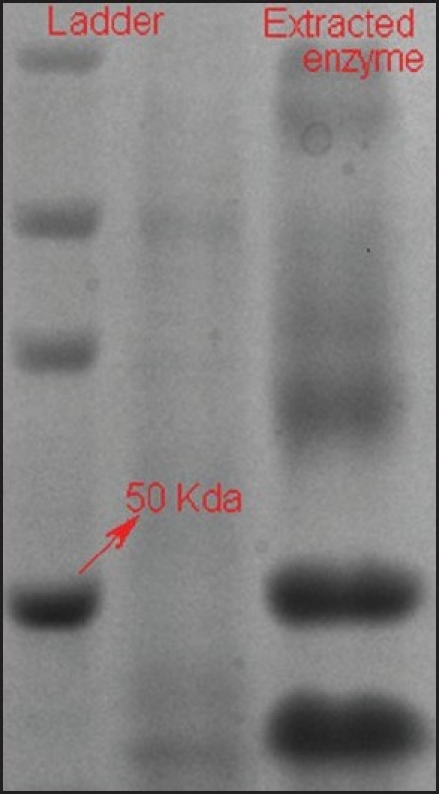
Determination of molecular weight of the enzyme by SDSPAGE electrophoresis The molecular size of the protease was determined by comparing it with the standard protein marker (15-150 KDa). The molecular weight of the bands was found to be 45.7 KDa and 38.5 KDa.

The next step was extraction of the enzyme and determination of the enzymic activity. Enzyme was obtained by ammonium sulfate salt precipitation and molecular size was determined by SDS-PAGE (fig. 3). Analysis showed two prominent bands. The molecular weight of the protease was determined by comparing it with the standard protein marker (15-150 KDa). The molecular weights of the bands were found to be 45.7 kDa and 38.5 kDa. Dahot has studied the properties of an alkaline protease from *Penicillum expansum* and reported the molecular weight of the enzyme to be 20.5 KDa[11]. Patke and Dey reported three proteases from *Streptomyces megasporus* in the range of 29 kDa to 90 kDa[12].

It is difficult to compare the enzyme activity as the conditions employed varied widely in the literature. Azeredo *et al*. produced a thermostable protease from a *Streptomyces* sp. 594 having an activity of 7.2 and 15.5 U/ml in submerged and solid-state fermentations, respectively[13]. The enzyme activity of PF_1_ was found to be 147.84 U/ml.

In the present study the cultural conditions for a protease producing fungal organism isolated from paddy soil of Manipal were optimized. Morphological studies indicate that the organism belonged to *Asperigillus niger* group of microorganisms. The enzyme extracted with ammonium sulfate showed two bands having mol.wts. 45.7 kDa and 38.5 kDa on SDS-PAGE and had an activity of 147.84 U/ml. Further work can be undertaken on enzyme characterization and the study can be extended to the pilot level for possible commercialization.
